# Species-level assessment of secondary metabolite diversity among *Hamigera* species and a taxonomic note on the genus

**DOI:** 10.1080/21501203.2014.917736

**Published:** 2014-05-27

**Authors:** Yasuhiro Igarashi, Tomoaki Hanafusa, Fumiya Gohda, Stephen Peterson, Gerald Bills

**Affiliations:** ^a^ Department of Biotechnology and Biotechnology Research Center, Toyama Prefectural University, 5180 Kurokawa, Imizu, Toyama939-0398, Japan; ^b^ US Department of Agriculture, National Center for Agricultural Utilization Research, 1815 North University Street, Peoria, IL61604, USA; ^c^ Texas Therapeutics Institute, The Brown Foundation Institute of Molecular Medicine, University of Texas Health Science Center at Houston, 1881 East Road, Houston, TX77054, USA

**Keywords:** chemotaxonomy, secondary metabolite, diversity, *Hamigera ingelheimensis*, Aspergillaceae, Eurotiomycetes, phylogeny

## Abstract

Secondary metabolite phenotypes in nine species of the *Hamigera* clade were analysed to assess their correlations to a multi-gene species-level phylogeny. High-pressure-liquid-chromatography-based chemical analysis revealed three distinctive patterns of secondary metabolite production: (1) the nine species could be divided into two groups on the basis of production of the sesquiterpene tricinonoic acid; (2) the tricinonoic acid-producing group produced two cyclic peptides avellanins A and B; (3) the tricinonoic acid-non-producing group could be further divided into two groups according to the production of avellanins A and B. The chemical phenotype was consistent with the phylogeny of the species, although metabolite patterns were not diagnostic at the species level. In addition, the taxonomy of the *Hamigera* clade was updated with the new combination *Hamigera ingelheimensis* proposed for *Merimbla ingelheimensis*, so that all species in the clade are now in the same genus.

## 1. Introduction

The Eurotiales include the important genera *Penicillium, Aspergillus, Talaromyces, Monascus, Paecilomyces* and other lesser known genera, e.g., *Hamigera*. Species of *Hamigera* are widespread and common soil fungi, and one species is associated with beetles (Peterson et al. 2010). A hallmark feature of many species of the Eurotiales is their rich secondary metabolism, which is evident by their biosynthesis of many harmful mycotoxins and important metabolites used as medicines and as chemical probes in cell biology. The genetic basis of this rich secondary metabolism is now known to be the result of the high number of secondary metabolite gene clusters present in their genomes. Some species, e.g., *Aspergillus nidulans* and *A. terreus*, have been estimated to have as many as 50–70 individual core biosynthetic genes for non-ribosomal peptide synthases (NRPS), polyketide synthases (PKS), terpene cyclases and prenyl transferases (Inglis et al. 2013).

Fungi of the Eurotiales have served as model species for developing chemosystematic classification methods in the higher fungi, a classification approach that employs data from standardized high-pressure liquid chromatography (HPLC) separation methods linked to UV and mass spectrometer (MS) detectors to generate profiles of metabolites characteristic of species and species complexes (Nielsen and Smedsgaard 2003; Frisvad et al. 2008). This approach of joining orthogonal data from metabolite patterns with morphology and phylogenetics to accurately diagnosis contaminating fungi has been pioneered at the Danish Technical University in conjunction with investigations of food, materials and environmental contaminants (Overy et al. 2003; Frisvad et al. 2004, 2007). The methods have been incorporated into extensive monographic works on many groups of *Aspergillus, Penicillium, Talaromyces* and *Monascus* and have consistently demonstrated that species definitions based on ribosomal and protein gene phylogenies correlate closely not only with the fungal morphological phenotype but also with the secondary metabolite phenotypes of individual strains belonging to a species (Frisvad and Samson 2004; Larsen et al. 2005; Frisvad et al. 2009; Nielsen et al. 2009; Samson et al. 2009; Slack et al. 2009). As of yet, an analysis of secondary metabolites has not been applied to the genus *Hamigera*.

**Figure 1.**
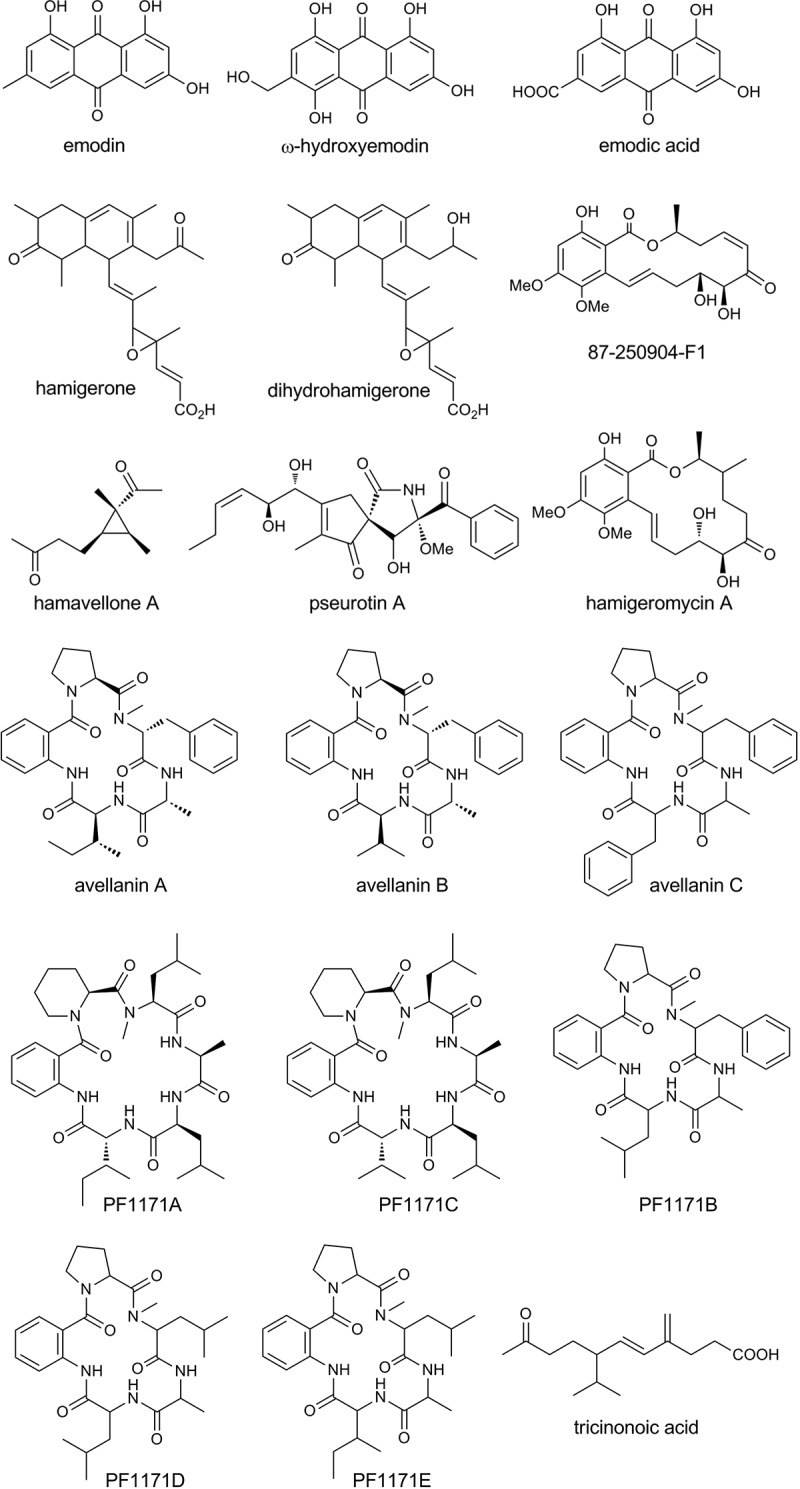
Secondary metabolites from *Hamigera* sp.

At least five metabolite families have been reported from *Hamigera avellanea* (Figure 1). The anthraquinone pigments emodin, ω-hydroxyemodin and emodic acid have been characterized from *H. avellanea* (Natori et al. 1965; Cehulová et al. 1996; Isaka et al. 2010). Hamigerone and dihydrohamigerone are polyketide antifungal metabolites (Breinholt et al. 1997). A compound known as 87-250904-F1 (=radicicol analogue A) is a resorcylic acid lactone that inhibits interleukin 1β and tumour necrosis factor-α secretion from the human monocytic leukemic cells (Mak et al. 2007) and has been found in *H. avellanea* along with a series of biosynthetically related nonaketide macrolactones named hamigeromycins A–G (Isaka et al. 2008, 2010). The same strain also produced a pair of novel cyclopropyl diketones designated hamavellones A and B, which were moderately toxic to *Plasmodium falciparum* and some human cancer cell lines. The strain co-produced pseurotin A, a competitive inhibitor of chitin synthase and an inducer of nerve cell proliferation, that is, the product of a hybrid NRPS–PKS gene cluster (Maiya et al. 2007). A family of cyclic hexapeptides and a pentapeptide, PF1171A-E, were described in a Japanese patent application from a strain of *H. avellanea* (FERM P-16173) (Umagome et al. 1999) for inhibition of the production of apolipoprotein B, the principal component of low-density lipoprotein. Based on amino acid sequences, these cyclic peptides are likely biosynthetically related to the pentapeptides avellanins A and B. Intravenous administration of avellanins caused vasoconstriction and increased blood pressure leading to death in mice (Yamazaki et al. 1987).

The genus *Hamigera* was recently revised by employing phylogenies constructed from rDNA and protein gene sequences. The analysis demonstrated that *Hamigera* comprises a monophyletic lineage of eight ascomata-forming species and one asexual species, with *H. avellanea* as the type species (Peterson et al. 2010). One of the main conclusions of this study was that the high variability observed in *H. avellanea* was the result of inadequate discrimination among a series of six newly recognized cryptic species. Furthermore, the asexual species *Merimbla ingelheimensis*, often thought to be the conidial state of *H. avellanea* was a distinct species within the *Hamigera* clade. The genus has recently been reclassified in the family Aspergillaceae and is a sister clade to *Aspergillus* and *Monascus* (Houbraken and Samson 2011).

This recent revision and the consolidation of a collection of all the newly described species from Europe, Asia, Africa, Micronesia, Australia, South America and North America at the Northern Regional Research Laboratory Culture Collection (NRRL) afforded an opportunity to test the hypothesis that these newly described taxa would contribute significant new variations in secondary metabolism beyond those previously reported compounds. In this report, representative strains of the nine species (one undescribed) were grown under a set of fermentation conditions, extracted with an organic solvent, and their extracts profiled by HPLC-diode array detector (DAD) to detect major UV-absorbing metabolites. We therefore describe the first genus-wide assessment of chemical diversity in *Hamigera* including reports of metabolites not previously encountered from species of this genus.

## 2. Materials and methods

### 2.1. Strains and phylogeny

Strains (Table 1) were obtained from the NRRL Collection and cultured on potato dextrose agar (PDA) at 20°C prior to generation of fermentation extracts. DNA sequences from three protein coding loci, *Mcm7, RPB2* and *Tsr1* (Peterson et al. 2010), were retrieved from GenBank (see Supplemental Table 1), and aligned using MUSCLE as implemented in MEGA5.2 (Tamura et al. 2011). The maximum likelihood tree of the *Hamigera* clade species was calculated using a GTR + G + I model in MEGA5.2. Bootstrap statistical support for the nodes were determined from 500 bootstrap iterations and are indicated on the tree. The tree diagram was formatted for publication using CorelDraw X6.

**Table 1.** Strains of the *Hamigera* clade examined for secondary metabolites.

Species	Strain number	Origin
*H. avellanea*	NRRL 1938	Soil, Texas, USA
*H. avellanea*	NRRL 58017	Soil, Amposta, Spain
*Hamigera* sp.	NRRL 2108	Rotting guayule, California, USA
*H. ingelheimensis*	NRRL 3522	Soil, India
*H. ingelheimensis*	NRRL 6221	Farm soil, Romania
*H. ingelheimensis*	NRRL 29060	Swine dung, Illinois, USA
*H. paravellanea*	NRRL 35714	Forest soil, Poland
*H. paravellanea*	NRRL 35720	Dung, Spain
*H. insecticola*	NRRL 35442	Coffee berry borer cuticle, Maryland, USA
*H. insecticola*	NRRL 35443	Coffee berry borer cuticle, Maryland, USA
*H. insecticola*	NRRL 58093	Indoor air, Indiana, USA
*H. inflata*	NRRL 58014	Soil, Gran Canary Island, Spain
*H. terricola*	NRRL 35602	Soil, Costa Rica
*H. terricola*	NRRL 35717	Soil, French Guiana
*H. terricola*	NRRL 35719	Soil, Equatorial Guinea
*H. pallida*	NRRL 35718	Soil, Togo
*H. fusca*	NRRL 29058	Soil, Australia
*H. fusca*	NRRL 35601	Soil, Australia
*H. fusca*	NRRL 35721	Soil, Grande Comore, The Comoros

### 2.2. Fermentations

Liquid inoculum was grown by inoculating agar plugs from PDA plates into 100 mL of medium V-22 (soluble starch 10 g, glucose 5 g, NZ-case 3 g, yeast extract 2 g, tryptone (Difco Laboratories) 5 g, K_2_HPO_4_ 1 g, MgSO_4_·7H_2_O 0.5 g and CaCO_3_ 3 g per litre distilled water; pH 7.0) and incubating flasks for 4-day growth at 30°C with agitation at 200 rpm. Aliquots of inoculum (3 mL) were transferred to 100-mL flasks containing six different production media including A-3M composed of glucose 0.5 g, glycerol 2 g, soluble starch 2 g, Pharmamedia (Archer-Daniels-Midland Co.) 1.5 g and yeast extract 0.3 g, per litre distilled water; super malt composed of malt extract 50 g, yeast extract 10 g, FeSO_4_·7H_2_O 2 mg and ZnSO_4_·7H_2_O 0.7 mg, per litre distilled water; A-11M composed of glucose 0.5 g, soluble starch 2.5 g, yeast extract 0.5 g, polypeptone 0.5 g, NZ-amine 0.5 g and CaCO_3_ 0.3 g, per litre distilled water; A-16 composed of glucose 2 g, Pharmamedia 1 g and CaCO_3_ 0.5 g, per litre distilled water; MGTY composed of maltose 15 g, glycerol 10 g, tryptone 10 g, yeast extract 10 g, KH_2_PO_4_ 1 g, MgSO_4_·7H_2_O 0.2 g and CaCl_2_ 0.5 g, per litre distilled water; SMY+A composed of maltose 40 g, peptone 10 g, yeast extract 10 g and agar 4 g, per litre distilled water. Production media were incubated 4 days at 30°C with agitation at 200 rpm.

### 2.3. Extraction and HPLC analysis

Equal volumes of 1-butanol were added to fermentations, and flasks were agitated for 60 min and then contents were centrifuged to separate fungal cells from the supernatant. A portion (1 mL) of the organic layer was concentrated under reduced pressure to give a crude extract that was then dissolved in dimethlysulfoxide (DMSO, 0.5 mL) for HPLC analysis on an Agilent HP1200 system equipped with a photodiode array detector. Spectral data were collected in the range from 200 to 600 nm. A measure of 100 μL of the DMSO solution was injected into an octadecylsilyl (ODS) column: HPLC condition A: Microsorb-MV^TM^ C-18 (Rainin Instrument, 3 μm, 4.6 × 75 mm), solvent was MeCN:0.15% KH_2_PO_4_ (pH 3.5) with the elution program as follows: 0–3 min (15% MeCN), 3–6 min (15–40% MeCN), 6–12 min (40% MeCN), 12–19 min (40–45% MeCN), 19–22 min (45–85% MeCN), 22–29 min (85% MeCN), 29–32 min (85–15% MeCN) with a flow rate of 1.2 mL/min, monitoring at 254 nm; HPLC condition B: XTerra^TM^ RP_18_, (Waters, 5 μm, 4.6 × 300 mm), solvent was MeCN:0.1% HCO_2_H at 40:60 ratio with a flow rate of 1.0 mL/min, monitoring at 254 nm.

### 2.4. Characterization of known and new compounds

The crude culture extract was fractionated by silica gel and ODS column chromatography and was further purified by preparative HPLC. Isolated compounds were subjected to spectroscopic analysis including nuclear magnetic resonance (NMR) and MS for structure determination.

## 3. Results and discussion

### 3.1. Taxonomy

*Hamigera ingelheimensis* S.W. Peterson, comb. nov., MB no. 807715

basionym: *Penicillium ingelheimensis* J.F.H. Beyma, Antonie van Leuuwenhoek 8:109, 1942; MB no. 289090.

≡ *Merimbla ingelheimensis* (J.F.H. Beyma) J.I. Pitt, Canadian Journal of Botany 57:2395, 1979; MB no. 317567.

≡ *Raperia ingelheimensis* (J.F.H. Beyma) Arx, Mycotaxon 26:121, 1986; MB no. 103783.

Holotype IMI 234977

Phylogenetically, *M. ingelheimensis* and *H. avellanea* are sister taxa, and concordance analysis of DNA sequences from multiple loci (Peterson et al. 2010) showed that they are distinct species. Under the auspices of article 59 from the nomenclatural code prior to 2012, the anamorphic *M. ingelheimensis* was placed in a distinct genus from its sister species *H. avellanea*. The Melbourne Code (Mcneill et al. 2012) changed the rules for naming pleiomorphic fungi and allowed only one valid name for a fungus, even if it had distinct morphologies that could occur independently and were named in different genera. All of the *Hamigera* species have a Merimbla-state in association with the teleomorphic state in *Hamigera. Hamigera ingelheimensis* has the Merimbla-state, but the teleomorphic state has not been observed. Because the Melbourne Code allows only a single name for a fungus and because intuitively we do not expect members of different genera to be more closely related that members of a single genus, we have formed the new combination *Hamigera ingelheimensis* to reflect the phylogenetic position of the species.

### 3.2. Secondary metabolite analysis

**Figure 2.**
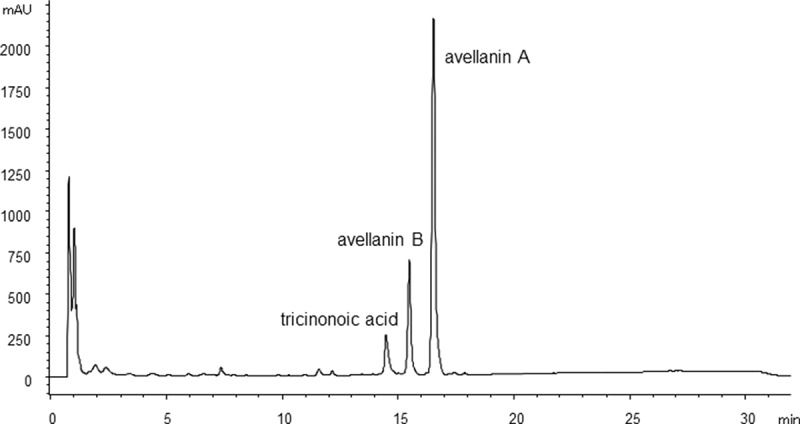
HPLC chromatogram of culture extract of *H. insecticola* NRRL35442 (shaking culture, A-16 medium, 4 days, HPLC condition A monitored at 254 nm).

**Figure 3.**
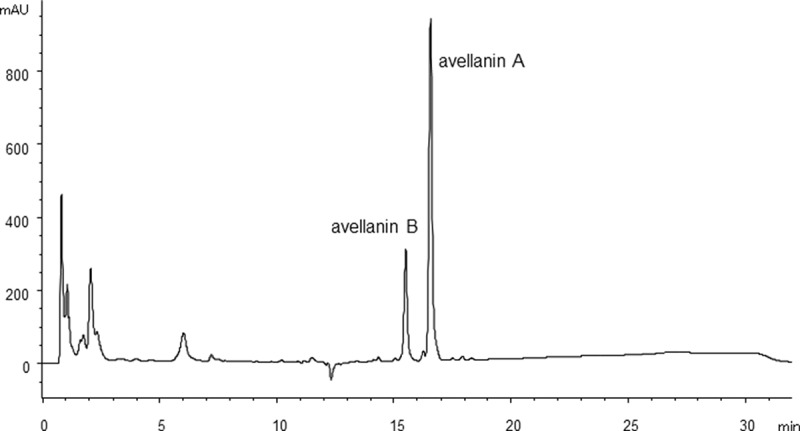
HPLC chromatogram of culture extract of *H. insecticola* NRRL35442 (static culture, A-16 medium, 21 days, HPLC condition A monitored at 254 nm).

Metabolite production was analysed by HPLC after 4, 6 or 10 days of cultivation with three strains (*Hamigera insecticola* NRRL35442, NRRL35443 and NRRL58093) in A-3M, A-11M and A-16 to establish the fermentation period for chemical phenotype pattern comparison. These strains produced tricinonoic acid and avellanins A and B as the major metabolites (Figure 2). Production of these three compounds was observed after 4 days, but no significant increase in production level was observed after 6 or 10 days of fermentation. Static culture was also tested but the period for production was substantially delayed (3–4 weeks) and did not significantly change metabolite patterns (Figure 3). Therefore, a 4-day fermentation period was fixed for the subsequent experiments.

Nineteen strains from the *Hamigera* clade (Table 1) were fermented in six different liquid media for 4 days on a rotary shaker. The whole culture broth was extracted with 1-butanol and the solvent extracts were analysed by HPLC-DAD. Major peaks were purified by consecutive fractionation on a silica gel and an ODS column chromatography, and final purification was done by preparative HPLC if necessary. The structures of isolated metabolites were analysed by using NMR, MS and UV techniques. From the 19 strains tested, 3 biosynthetically different classes of metabolites were identified (Table 2): tricinonoic acid, which is a sesquiterpene; avellanins and PF1171s, which are cyclic pentapeptides and hexapeptides; and anthraquinones, which are aromatic polyketides.

**Table 2.** Distribution of tricinonoic acid and peptide metabolites in *Hamigera* species.

Species and NRRL strain number	Tricinonoic acid	Avellanins A & B	PF1171B	Avellanin C*	PF1171A	PF1171C	Anthraquinone**
Structural class	Sesquiterpene	Pentapeptides			Hexapeptides		Polyketide
*H. avellanea 1938*	*–*	○	○	–	–	–	*–*
*H. avellanea 58017*	*–*	○	○	–	–	–	–
*Hamigera* sp. *2108*	*–*	○	–	–	–	–	–
*H. ingelheimensis 3522*	*–*	–	–	–	–	○	–
*H. ingelheimensis 6221*	*–*	–	–	–	–	–	–
*H. ingelheimensis 29060*	*–*	–	–	○	–	–	–
*H. paravellanea 35714*	*–*	○	○	○	○	○	○
*H. paravellanea 35720*	*–*	○	○	–	–	○	–
*H. insecticola 35442*	*○*	○	–	–	–	–	–
*H. insecticola 35443*	*○*	○	–	–	–	–	–
*H. insecticola 58093*	*○*	○	–	–	–	–	–
*H. inflata 58014*	*○*	○	–	–	–	–	–
*H. terricola 35602*	*○*	○	–	–	–	–	–
*H. terricola 35717*	*○*	○	–	–	–	–	–
*H. terricola 35719*	*○*	○	–	–	–	–	–
*H. pallida 35718*	*○*	○	–	–	–	–	–
*H. fusca 29058*	*○*	○	○	–	–	○	–
*H. fusca 35601*	*○*	○	○	–	–	○	–
*H. fusca 35721*	*–*	–	–	–	–	–	–

Notes: *New compound.

**Aromatic polyketide (UV *λ*_max_ = 445 nm); structure not determined.

**Figure 4.**
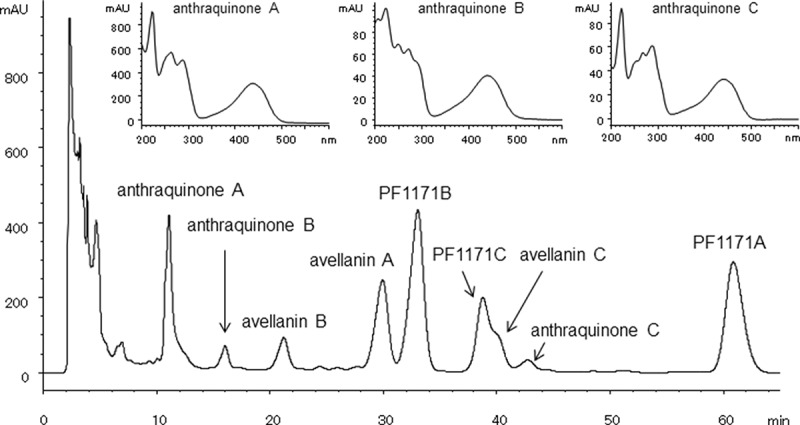
HPLC chromatogram of culture extract of *H. paravellanea* NRRL35714 (shaking culture, supermalt, 4 days, HPLC condition B monitored at 254 nm).

**Figure 5.**
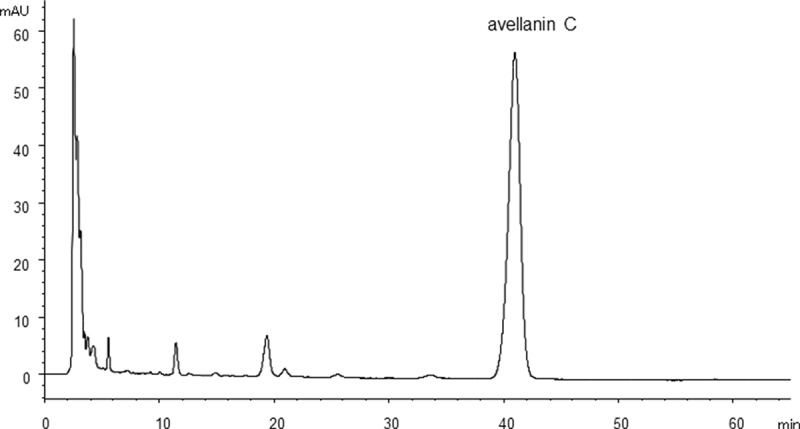
HPLC chromatogram of culture extract of *H. ingelheimensis* NRRL29060 (shaking culture, A-3M, 4 days, HPLC condition B monitored at 254 nm).

The most commonly produced metabolites were avellanins A and B (Table 2). Fifteen of the 19 strains representing 8 of the 9 species produced these cyclic pentapeptides. Three strains of *H. ingelheimensis* did not. The second most common metabolite was tricinonoic acid, a sesquiterpene first isolated from *Fusarium tricinctum* (Bashyal and Leslie Gunatilaka 2010). To the best of our knowledge, production of tricinonoic acid has not been reported from *Hamigera* or *Merimbla*. The production pattern of this terpene compound divided nine *Hamigera* species into two groups. The producer group contains *H. insecticola, H. inflata, H. terricola, H. pallida* and *H. fusca*, and the non-producer group containing *Hamigera* sp., *H. avellanea, H. ingelheimensis* and *H. paravellanea*. Interestingly, all three *H. ingelheimensis* strains produced neither avellanins A and B or tricinonoic acid. Avellanins A and B, first reported from *H. avellanea*, consist of five amino acid components: alanine, phenylalanine, proline and anthranillic acid as common monomers and isoleucine for avellanin A and valine for avellanin B. PF1171B, the leucine version of the pentapeptide avellanin A was produced only by three species, *H. avellanea, H. paravellanea* and *H. fusca*. PF1171A and PF1171C are the cyclic hexapeptides first isolated from *H. avellanea*. The major difference of these peptides from avellanins is the substitution of pipecolic acid for proline. Production of PF1171C was limited to three species, *H. ingelheimensis, H. paravellanea* and *H. fusca. H. paravellanea* NRRL35714 produced another hexapeptide PF1171A and a new pentapeptide avellanin C in which the isoleucine residue of avellanin A was replaced by phenylalanine (Figure 4). Details on structure determination and biological properties of avellanin C will be reported in our forthcoming paper. It is noteworthy that avellanin C was also produced by *H. ingelheimensis* NRRL29060 and was the only compound detected from this strain (Figure 5). Production of anthraquinone-class polyketides that displayed typical UV λ_max_ spectral profile with the absorption maximum around 445 nm (Figure 4) was noticed only with *H. paravellanea* NRRL35714 although their structures were not fully elucidated yet. Strain NRRL35714 was the most productive strain tested in this study, which produced the full range of cyclic pentapeptides and hexapeptides and aromatic polyketides.

Genomic sequencing has not been completed for any *Hamigera* species to date; therefore, data on genome size and numbers and distribution of secondary metabolite gene clusters in these fungi remain unknown. Based on secondary metabolome studies of other species of the Eurotiales, it is likely that we have only sampled a portion of the secondary metabolites encoded in the genomes of these fungi. Our assessment is preliminary because we have not exhaustively probed media and fermentation techniques for stimulating metabolite expression, and our primary detection method was based on UV absorbance. Perhaps, mass-based detection methods or sensitive biological assays would have revealed additional metabolite complexity.

**Figure 6.**
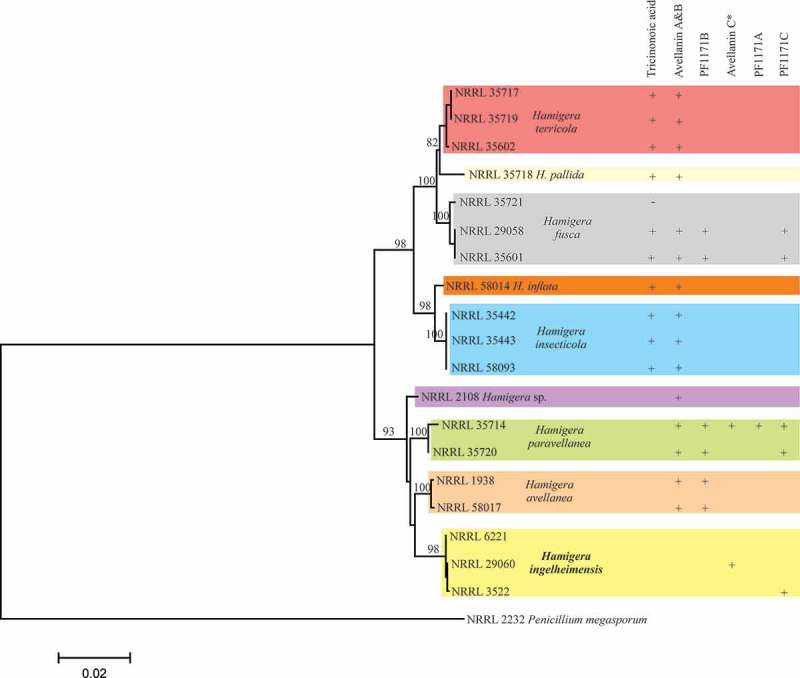
Phylogenetic tree showing the relations of *Hamigera* species.

Our data did not provide species-level diagnostic patterns. Nonetheless, the major metabolites reported here were consistent with the phylogeny of the species; tricinonoic acid production was a definitive character for the clade containing *H. terricola, H. pallida, H. fusca, H. inflata* and *H. insecticola* (Figure 6). Other metabolites such as PF1171B and PF1171C were variably present in strains of species from each of the main clades of *Hamigera*. This pattern could be explained by presence of their pathways in the most recent common ancestor followed by loss of function in some descendent species or simply lack of expression in some of the strains and species during our study. Most studies (e.g. Frisvad and Samson 2004) that use chemotaxonomy as part of the identification scheme use a broader range of metabolites to reliably identify species. That would appear to be the case in *Hamigera* also. Statistically convincing chemotaxonomy must await a more thorough mining of the secondary metabolites produced by the species and isolates and use of more chemical markers.
